# From Motivation to Organizational Identity of Members in Non-profit Organizations: The Role of Collectivism

**DOI:** 10.3389/fpsyg.2020.01881

**Published:** 2020-07-31

**Authors:** Yong Li, Yuting Zhang

**Affiliations:** ^1^School of Marxism, Shanghai Maritime University, Shanghai, China; ^2^School of Management, Zhejiang University of Technology, Hangzhou, China

**Keywords:** altruism, egoism, collectivism, organizational identity, NPO

## Abstract

This study contributes to our understanding of organizational identity through dichotomous motivations of altruism and egoism in non-profit organizations (NPO). By applying an empirical analysis of NPO members, organizational identity is found to be well explained by altruistic motivation and egoistic motivation. More importantly, this study finds that collectivism positively moderates the relationship between altruistic motivation and organizational identity, and negatively moderates the relationship between egoistic motivation and organizational identity. It is noticeable that altruistic motivations have a stronger impact on organizational identity when collectivism is high, while egoistic motivations have a stronger impact on organizational identity when collectivism is low. Finally, this study generates helpful management implications based on research findings. It is suggested that the managers of NPOs could enhance members’ organizational identity by taking motivations and collectivism into consideration, that is to say, in order to build up organizational identity of NPO members, both righteousness and shared interests matter simultaneously.

## Introduction

Non-profit organizations (NPO) have grown tremendously in China over the past two decades (Ma and Liu, 2019). Correspondingly, the focus to human resource management (HRM) in NPOs has aroused more scholarly interest over the last few years (Akingbola, 2013b; Baluch et al., 2013; Kellner et al., 2017). HRM practices are associated with high organizational identity and low quit rates amongst organization members, so understanding HRM practices is essential to the development of NPOs. What is particular about NPOs is that such organizations are designed to achieve social outcomes rather than generating profit (Kong, 2008; Surtees et al., 2014), while volunteers in NPOs often lack of economical rewards (Lynn, 2003). Given the fundamental differences in the missions, value-orientation, and financial restrictions of non-profit and private organizations, there are obvious differences between HRM in NPOs and HRM in profit organizations (Ban et al., 2003; Ridder and McCandless, 2010; Akingbola, 2013a). Therefore, performance-oriented approach to HRM may conflict with the mission and values of NPOs. Some studies indicate that NPO members seemingly have self-perpetuating commitment regardless of wages and working conditions along with their astounding resilience and ability to tolerate increasing levels of stress at the workplace (Baines, 2006; Nickson et al., 2008; Baines, 2010). NPOs often put a stronger emphasis on employees’ altruism motivations, whereby the mission becomes an effective tool for fostering motivation, retention and maintaining members’ organizational identity (Brown and Yoshioka, 2003; Kim and Lee, 2007).

For the growth of an organization, organizational identity serves as the main way for organizations to enhance cohesion and plays a key role in organizations’ success (Mael and Tetrick, 1992; Reade, 2001; Smidts et al., 2001). Likewise, organizational identity is one of the goals pursued by the NPO human resources management. High organizational identity could help improve organization’s operation efficiency and resilience, and promote its sustainable development. Moreover, the organizational identity’s positive influence is of great significance to organization featured by long-lasting emotional bond (Gioia et al., 2000). By contrast, lack of organizational identity may lead to serious organization problems, such as members’ turnover and organization failure (Kreiner and Ashforth, 2004). Therefore, building up organizational identity has become one key task for NPO development in the modern world.

Motivation is found to be vital for organizational identity (Gioia et al., 2013). The occupational choice of individuals originates from an intricate mixture of motivations. For egoism, the ultimate goal is to increase one’s own welfare; for altruism, it is to increase the welfare of another individual or the public (Batson et al., 2002). With the constant development of NPO practice, NPOs have exerted broader influence and attracted more and more members. Likewise, the motivations of NPO members can be mainly divided into two types, of which, one is the attempt to resolve social problems and assistance for others. The other is the potential employment and overseas study opportunity that NPO can provide, or the pursuit of a favorable reputation, or access to volunteer service certificate and scholarship. Yet, the evidence on the relationship between motivation and organizational identity remains insufficient for NPO.

Therefore, this study explores the shaping mechanism of altruistic motivation and egoistic motivation on the organizational identity of NPO members respectively. Along with the fast development of economy, China’s occupational values has been changed profoundly which can be reflected by the new generation of Chinese who have more individualism value and less collectivism value than the previous generation (Ralston et al., 1996). Furthermore, this study examines the direct effect of collectivism, and moderating effect of collectivism on the relationship between altruism and organizational identity, egoism and organizational identity. This study aims to contribute to the literature on the following two aspects: (1) It deepens our understanding of the mechanism from motivation to organizational identity under the context of NPO. (2) It provides suggestions for NPO managers to enhance their members’ organizational identity, which bring critical practice significance to NPO’s human resources management and sustainable development.

**FIGURE 1:**
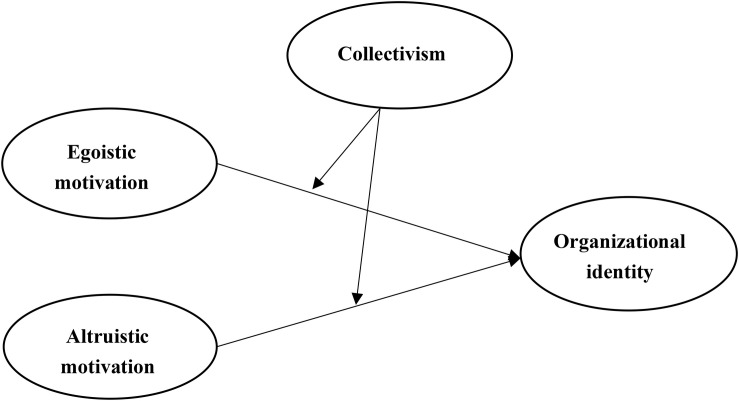
Hypothesized model.

Figure 1 shows the hypothesized model of this study.

## Literature Review and Hypotheses

### Altruism, Egoism, and Organizational Identity

Organizational identity has long been recognized as a critical construct of organizational behavior, affecting both the satisfaction of the individual and the effectiveness of the organization (Hall et al., 1970; Lee, 1971; Ashforth and Mael, 1989). Organizational identity refers to individual’s perception of belonging to the organization. To be specific, a member with a high organizational identity has group consciousness, and believes he/she is an inseparable part of the organization.

The rational incentive hypothesis indicates that self-interest is at the root of human behavior. Human behavior is motivated primarily by self-interest (Perry and Wise, 1990). However, the rational incentive theory based on the maximization of personal interests cannot best understand and guide the NPO’s practice. NPO features as voluntariness purpose. In general, the members of NPO are inclined to provide benefits such as social concern and helpful behaviors (Lester et al., 2008). In view of this, the altruistic motivation should be emphasized for the influence on the organizational identity under the context of NPO. Indeed, the motivation for the shaping of organizational identity is multidimensional, not one-dimensional. Both altruism and egoism are vitally motivational aspects. Altruistic motivation indicates that people choose to join in NPO because they have compassion and want to contribute to public interest. Egoistic motivation, in contrast, involves people choosing to be a member of NPO because of tangible or intangible rewards which the choice leads (Gagné and Deci, 2005). Members with high altruistic motivation share the same goal with the NPO and take the NPO as the social service platform. As a result, they will exert their devotion enthusiasm and propel the organization to make achievements. On the contrary, members with high egoistic motivation take NPO as a platform for potential benefits and will enhance their personal organizational identity in order to make their resume outstanding when applying for a job or study abroad opportunity in the future. Accordingly, we predict that altruistic motivation and egoistic motivation positively influence the organizational identity of NPO members.

Hypothesis 1a: Altruistic motivation has a positive impact on organizational identity.
Hypothesis 1b: Egoistic motivation has a positive impact on organizational identity.

### The Moderating Role of Collectivism

Collectivism value, as one of the cultural values that have received widespread attention and heated discussion, reflects the degree of concern of individuals on other individuals and organization (Hui, 1988; Felfe et al., 2008). Compared with the members with low collectivism who lay emphasis on personal interests and are indifferent to the organization (Hofstede, 1984), the members with high collectivism could regard themselves as part of the collective and always believe collective interest outweighs individual interests (Hui, 1988). In other words, members with high collectivism are in a social network that links everyone closely and highlights the loyalty and emotional dependence on the organization.

Concretely speaking, we will discuss the contingency influence of members’ collectivism value on the relationship between altruistic motivation and organizational identity, and the relationship between egoistic motivation and organizational identity, respectively. It is noticeable that the collectivist tends to distinguish others into in-group and out-group, and is willing to share the personal knowledge and information with those in group that he/she is familiar with (Triandis, 1995; Voelpel and Han, 2005). Therefore, when concerning the pattern from altruistic motivation to the organization identity of NPO members, the high collectivism will intensify the positive function of altruism on organizational identity. In other words, those with high collectivism are inclined to the collective and hope to grow together with the NPO they work for. Besides, altruism and NPOs’ missions are perfectly compatible with each other. Therefore, both the collectivism and altruistic motivation mutually enhance the organizational identity. By contrast, when concerning the pattern from egoistic motivation to the organization identity of NPO members, the high collectivism may lose its role. For those with low collectivism who do not lay emphasis on the integrity of individuals with organization, they join in NPO for instrumental purpose clearly, which means they want the potential employment or study abroad opportunity that NPO could bring. That is to say, NPO members with low collectivism can only get identification from instrumental rewards rather than emotional dependency that binds individuals to organizations. Therefore, the degree of organizational identity shaped by egoistic motivation is more prominent under low collectivism. Thus, the following hypotheses are proposed.

Hypothesis 2a: The positive relationship between altruistic motivation and organizational identity will be stronger as collectivism is higher.
Hypothesis 2b: The positive relationship between egoistic motivation and organizational identity will be stronger as collectivism is lower.

## Materials and Methods

### Sample and Data Collection

The whole survey that lasted for about 2 months was conducted in NPOs in China. Before the formal date of collection, we carried out a pilot testing with a sample of 20 NPOs to examine whether the overall survey was clear, thorough, and robust. After the pilot test, the investigators modified the wording of some items according to the feedback from the participants. The formal survey was conducted in Zhejiang and Sichuan Provinces. Members of NPOs participated in the study on a voluntary basis, and a convenience sampling was adopted. Overall, 68 NPOs were involved. There are totally 130 questionnaires given out and 114 received. All the participants are volunteers of NPOs. We conducted the hierarchical regressions on SPSS 20.0 to test the hypotheses proposed in this study.

### Common Method Bias

As we asked the same individual about both dependent and independent variables at the same time, the common method bias (CMB) should be concerned (Podsakoff et al., 2003). In order to reduce the risk of CMB, following steps are adopted. First, we guaranteed individuals’ anonymity and we distributed the questions for dependent and independent variables intentionally, which could minimize the CMB on the procedure (Krishnan et al., 2006). Second, the Harman’s one-factor method was performed to test the potential problems of CMB (Podsakoff et al., 2003). If one factor could account for most of the covariance among the variables, CMB would exist. In this study, the unrotated exploratory factor analysis demonstrated that the first factor explained less than 40% of variance. This level of variance cannot invalidate the relationships between independent and dependent variables (Doty and Glick, 1998; Fuller et al., 2016), confirming no serious issue of CMB (Podsakoff et al., 2003).

### Measurement

This study aims to propose a model of how altruistic motivation and egoistic motivation affect organizational identity of NPO members under different levels of collectivism. The main variables are measured as follows.

The dependent variable. Organizational identity was measured by the 6-item scale developed by Mael and Ashforth (1992). A Likert Scale was used for evaluation, in which 1 means “disagree strongly,” and 5 means “agree strongly.” The sampling question is “It feels like a personal insult when somebody criticizes my serving organization.”

The independent variables. Altruism motivation was measured by the 8-item scale developed by Perry (1996). A Likert Scale was used for evaluation, in which 1 means “disagree strongly,” and 5 means “agree strongly.” The sampling question is “Making a difference to society means more to me than personal achievements”. The Egoistic motivation was measured by the 4-item scale developed by Wrzesniewski et al. (2014). A Likert Scale was used for evaluation, in which 1 means “disagree strongly,” and 5 means “agree strongly.” The sampling entry is “I can get a better job after I join my serving organization.”

The moderated variable. Collectivism was measured by the 5-item scale developed by Earley (1993). A Likert Scale was used for evaluation, in which 1 means “disagree strongly,” and 5 means “agree strongly.” The sampling question is “A member should accept the group’s decision even when personally he or she has a different opinion.”

Control variables. Apart from demographic variables such as gender, age, education, tenure (Mael and Ashforth, 1992) was also applied as control variable in the data analysis.

## Results

### Descriptive Analyses

Table 1 shows descriptive statistics of the sample, in which there are more men than women. The participants’ age ranges from 18 to 65. Table 1 also shows that most participants have a Bachelor degree. Most participants’ tenure is less than 5 years. Table 2 presents the descriptive statistics resulting from our analysis, including means, standard deviations, and a correlation matrix. Consistent with our hypotheses, the correlations among altruistic motivation, egoistic motivation, collectivism, and organizational identity were all significant. The Cronbach’s α of each dimension was greater than 0.80, indicating good reliability (Nunnally, 1978). The composite reliability (CR) of latent variables are all greater than 0.60, indicating that the variables have good internal consistency (Fornell and Larcker, 1981). The average variance extracted (AVE) of variables are range from 0.51 to 0.74, which is higher than the benchmark of 0.3 recommended by Fornell and Larcker (Fornell and Larcker, 1981).

**TABLE 1:** Demographic information of the sample.

Demographic variable	Percentage
**Gender**	
Male	56
Female	44
**Age**	
18–30	61
31–65	39
**Education**	
Pre-college student	12
Bachelor	76
Graduate or above	12
**Tenure**	
Less than a year	49
One year to 5 years	39
More than 5 years	12

**TABLE 2:** Descriptive statistics and correlations.

No.	Variables	Mean	*SD*	Cronbach’s alpha	CR	AVE	1	2	3	4	5	6	7	8
1	Gender	0.58	0.51				1.00							
2	Age	30.27	8.07				–0.01	1.00						
3	Education	1.99	0.49				0.16	–0.36	1.00					
4	Tenure	28.17	33.07				–0.15	0.60**	–0.30	1.00				
5	Altruistic motivation	3.78	0.76	0.84	0.89	0.62	–0.23	0.27**	–0.18	0.20*	1.00			
6	Egoistic motivation	3.49	0.87	0.83	0.90	0.74	–0.04	–0.01	0.07	–0.08	0.33**	1.00		
7	Collectivism	4.04	0.69	0.84	0.88	0.59	–0.13	0.52**	–0.20	0.30**	0.47**	0.20*	1.00	
8	Organizational identity	3.83	0.72	0.81	0.86	0.52	0.08	0.31**	–0.09	0.27**	0.45**	0.54**	0.40**	1.00

**p < 0.01, *p < 0.05, n = 114.

### Testing Hypotheses

As a tentative exploration of the effect of altruistic motivation, egoistic motivation and collectivism on organizational identity, we conducted ordinary least square regressions, involving three models in total. The results of regression models are presented in Table 3. Hypothesis 1a and Hypothesis 1b proposed that altruistic motivation and egoistic motivation had positive impact on organizational identity, which was verified by Model 2 (β = 0.256, *p* < 0.001; β = 0.397, *p* < 0.001). The results in Model 3 showed that collectivism positively moderated the relationship between altruistic motivation and organizational identity (β = 0.110, *p* < 0.005), and negatively moderated the relationship between egoistic motivation and organizational identity (β = -0.180, *p* < 0.001). Therefore, Hypothesis 2a and Hypothesis 2b were fully supported.

**TABLE 3:** Results of regression analysis.

	Organizational identity		
	
	Model 1	Model 2	Model 3
**Control variables**			
Gender	0.138	0.264*	0.274**
	(0.133)	(0.105)	(0.102)
Age	0.020^†^	0.010	–0.001
	(0.011)	(0.008)	(0.009)
Education	0.027	–0.005	0.005
	(0.145)	(0.114)	(0.108)
Tenure	0.003	0.005*	0.005*
	(0.003)	(0.002)	(0.002)
**Independent variables**			
Altruistic motivation		0.256***	0.237**
		(0.077)	(0.078)
Egoistic motivation		0.397***	0.383***
		(0.063)	(0.060)
**Moderator variable**			
Collectivism			0.167^†^
			(0.091)
Altruistic motivation^∗^Collectivism			0.110*
			(0.049)
Egoistic motivation^∗^Collectivism			−0.180***
			(0.055)
Constant	3.002***	0.911*	0.624
	(0.462)	(0.462)	(0.451)
Adjusted *R*^2^	0.084	0.443	0.501
*F*-value	3.555**	15.874***	13.518***
Sample	114	114	114

***p<0.001, **p<0.01, *p<0.05, ^†^p < 0.1.

**FIGURE 2:**
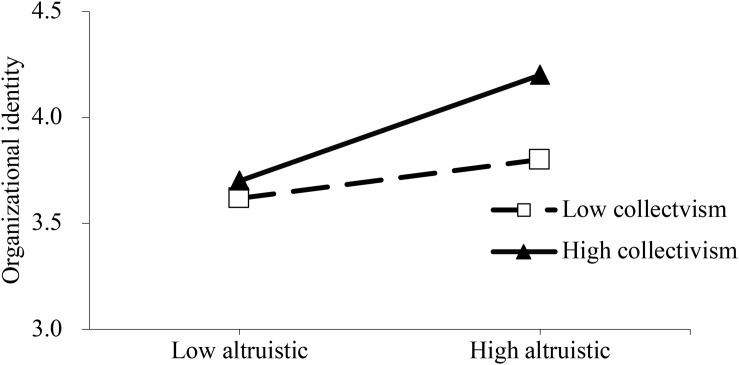
Altruistic motivation and organizational identity – the moderating role of collectivism.

**FIGURE 3:**
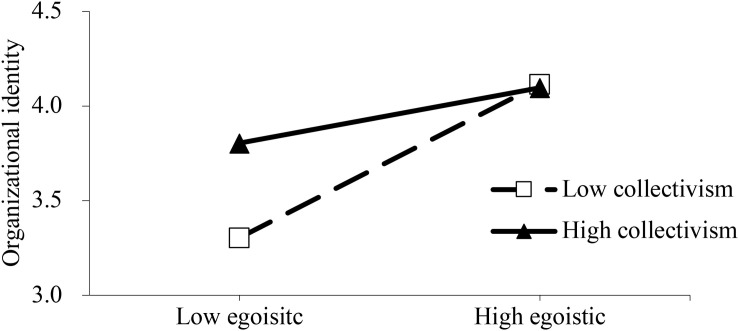
Egoistic motivation and organizational identity – the moderating role of collectivism.

In order to more clearly characterize the moderation mechanism, slope tests were conducted to evaluate whether the relationship between altruistic motivation and organizational identity, and the relationship between egoistic motivation and organizational identity were intensified or weakened by different levels of collectivism. Figures 2, 3 show that NPO members with high collectivism has stronger organizational identity in NPO. More importantly, Figure 2 illustrates that collectivism could intensify the positive effect of altruistic motivation on organizational identity. Figure 3 illustrates that collectivism could weaken the positive effect of egoistic motivation on organizational identity.

## Limitations

This study has some limitations that may require further discussion and exploration. First, since cross-section data were used, it was unable to efficiently judge the causality between variables. Thus, in a subsequent study, the methods of longitudinal and cross-level research can be further applied to make the outcome more perfect and enhance the quality. Second, the sample size of our study is relatively small which may in turn effect the reliability of the results. Future study may involve more NPOs across several provinces in China so that more comprehensive data can be collected.

## Discussion

The past two decades have witnessed an unprecedented boom in the NPOs in China (Ma and Liu, 2019). Accordingly, the human resource management of NPOs and organizational identity promotion of NPO members have been paid more and more attention (Buonomo et al., 2020). Organizational identity is a stabilizing force that binds individuals to organizations (Ng, 2015). In view of the continuity of organizational identity, members who have resigned from the organization will still behave in a way that benefits the organization. This study sheds light on the “black box” of how diverse motivations and collectivism value lead to the organizational identity of NPO members.

The research findings of this study show that both altruistic and egoistic motivations exert significantly positive influence on the organizational identity of NPO members. This indicates that altruism and egoism are not dichotomous, and they jointly matter in the shaping organizational identity in the context of NPOs. In other word, to join in NPOs involves righteousness and shared interests simultaneously. Furthermore, the collectivism value could exert roles of reverse moderating on the relationships between motivations and organizational identity. For the members with high collectivism, altruistic motivation is the key source of organizational identity. However, for the members with low collectivism, egoistic motivation become a key source of organizational identity. On the era of the declining collectivism value (Ralston et al., 1996), it is suggested that the managers of NPOs should update their idea about building up organizational identity, shifting from a focus on members’ altruism to a balance of altruism and egoism wherein individuals choose to engage in NPOs to garner future benefits. In order to enhance the organizational identity of NPO members, this study suggests that the managers of NPOs should not only lay emphasis on the guidance of altruism and collectivism value but also design diversified incentive ways.

Furthermore, the study is useful to NPO managers aiming to develop targeted approaches to attracting and retaining volunteers. In view of the above conclusions, the following optional methods can be adopted to promote the human resource management for NPO members. First, emphasizing the mission and values of NPOs through HR practices such as recruitment, selection, orientation and training is an important tool for ensuring motivation and retention of members (Brown and Yoshioka, 2003; Kim and Lee, 2007). This study suggests that the manager of NPO could promote understanding members’ motives, expectations of the organization in the early stage. This is a prerequisite for effective incentives and building organizational identity. It is necessary for NPO managers to have priority in recruiting the candidates with high altruism and collectivism in the process of personnel recruitment, and have priority in cultivating them as the core members for management positions. Only by conducting this strategy can the NPO realize the organization’s mission and shared vision to the maximum in the long term. Nowadays, plenty of the NPOs are under growth stage and still need some time before becoming mature. When the growing NPOs face challenges, the members with high altruism and collectivism will unite with each other closely, and believe they are largely tied with the organization’s destiny (Epitropaki and Martin, 2005). Such kind of members will ride out a storm together with the organization. Second, this study suggests that the NPO managers can arrange executed work for high egoism members, so as to optimize human resource allocation. Regarding the members with high egoistic motivation, systematic performance evaluation means, and timely incentive and awarding measures are necessary for them to build mutual trust and cooperation willingness, which will contribute to their higher organizational identity (Malhotra and Murnighan, 2002). Third, increasing investment in training and delivering organizational mission can be viewed as effective measures to attract and retain volunteers in NPOs. Regarding to enhancing volunteer organizational identity, this study suggests that the manager of NPO need attach importance to organizational support, and the HR practices of NPOs should signal to volunteers in a long-term, mutual relationship, viewing volunteers as valuable resources.

## Conclusion

To conclude, in order to build up organizational identity of NPO members, it is noticeable that righteousness and shared interests simultaneously matters. Thus, the view of shifting from a focus on altruism to a balance of altruism and egoism has stronger explanatory power for the shaping of NPO members’ organizational identity. Based on our research result, it is suggested that NPOs should balance values-based HRM system and performance-based HRM system.

## Data Availability Statement

The raw data supporting the conclusions of this article will be made available by the authors, without undue reservation, to any qualified researcher.

## Ethics Statement

Ethical review and approval was not required for the study on human participants in accordance with the local legislation and institutional requirements. Written informed consent for participation was not required for this study in accordance with the national legislation and the institutional requirements.

## Author Contributions

All authors contributed equally to formulating the conceptual framework, analyzing the data, and writing the manuscript.

## Conflict of Interest

The authors declare that the research was conducted in the absence of any commercial or financial relationships that could be construed as a potential conflict of interest.
